# Exoskeleton for post-stroke recovery of ambulation (ExStRA): study protocol for a mixed-methods study investigating the efficacy and acceptance of an exoskeleton-based physical therapy program during stroke inpatient rehabilitation

**DOI:** 10.1186/s12883-020-1617-7

**Published:** 2020-01-28

**Authors:** Dennis R. Louie, William B. Mortenson, Melanie Durocher, Robert Teasell, Jennifer Yao, Janice J. Eng

**Affiliations:** 1 Graduate Program in Rehabilitation Sciences, Faculty of Medicine, University of British Columbia, Vancouver, British Columbia Canada; 2 Rehabilitation Research Program, Vancouver Coastal Health Research Institute, 212-2177 Wesbrook Mall, Vancouver, BC V6T 1Z3 Canada; 3 Department of Occupational Science and Occupational Therapy, Faculty of Medicine, University of British Columbia, Vancouver, British Columbia Canada; 4 Glenrose Rehabilitation Hospital, Alberta Health Services, Edmonton, Alberta Canada; 5 Parkwood Institute Research, Lawson Health Research Institute, London, Ontario Canada; 6 Parkwood Institute, St Joseph’s Health Care London, London, Ontario Canada; 7 Schulich School of Medicine & Dentistry, Western University, London, Ontario Canada; 8 Division of Physical Medicine and Rehabilitation, Faculty of Medicine, University of British Columbia, Vancouver, British Columbia Canada; 9 GF Strong Rehabilitation Centre, Vancouver Coastal Health, Vancouver, British Columbia Canada; 10 Department of Physical Therapy, Faculty of Medicine, University of British Columbia, 212-2177 Wesbrook Mall, Vancouver, BC V6T 1Z3 Canada

**Keywords:** Stroke, Rehabilitation, Exoskeleton, Walking, Clinical trial

## Abstract

**Background:**

The ability to walk is commonly reported as a top rehabilitation priority for individuals after a stroke. However, not all individuals with stroke are able to practice walking, especially those who require more assistance from their therapist to do so. Powered robotic exoskeletons are a new generation of robotic-assisted gait training devices, designed to assist lower extremity movement to allow repetitious overground walking practice. To date, minimal research has been conducted on the use of an exoskeleton for gait rehabilitation after stroke. The following research protocol aims to evaluate the efficacy and acceptability, and thus adoptability, of an exoskeleton-based gait rehabilitation program for individuals with stroke.

**Methods:**

This research protocol describes a prospective, multi-center, mixed-methods study comprised of a randomized controlled trial and a nested qualitative study. Forty adults with subacute stroke will be recruited from three inpatient rehabilitation hospitals and randomized to receive either the exoskeleton-based gait rehabilitation program or usual physical therapy care. The primary outcome measure is the Functional Ambulation Category at post-intervention, and secondary outcomes include motor recovery, functional mobility, cognitive, and quality-of-life measures. Outcome data will be collected at baseline, post-intervention, and at 6 months. The qualitative component will explore the experience and acceptability of using a powered robotic exoskeleton for stroke rehabilitation from the point of view of individuals with stroke and physical therapists. Semi-structured interviews will be conducted with participants who receive the exoskeleton intervention, and with the therapists who provide the intervention. Qualitative data will be analyzed using interpretive description.

**Discussion:**

This study will be the first mixed-methods study examining the adoptability of exoskeleton-based rehabilitation for individuals with stroke. It will provide valuable information regarding the efficacy of exoskeleton-based training for walking recovery and will shed light on how physical therapists and patients with stroke perceive the device. The findings will help guide the integration of robotic exoskeletons into clinical practice.

**Trial registration:**

NCT02995265 (clinicaltrials.gov), Registered 16 December 2016.

## Background

Stroke is a leading cause of adult disability, often resulting in hemiparesis, altered sensation, incoordination, cognitive changes, and speech disturbances [1, 2]. With improved detection and medical treatment of stroke, the prevalence of individuals living with such effects of stroke is continually increasing at the national and global scale [2–4]. A major factor associated with long-term disability after stroke is the ability to walk independently [5–8], and is often cited as a goal by individuals with stroke [9, 10]. However, nearly half of individuals with stroke do not regain the ability to walk independently, even after rehabilitation [11, 12]. It is thus important to develop rehabilitation strategies that will promote walking recovery after stroke.

Current best practice guidelines recommend that individuals with stroke should engage in early rehabilitative training that is intensive, repetitive, and task-specific to improve mobility and walking [13, 14]. However, it can be challenging to reach this guideline for individuals with more severe stroke; the amount of walking practice achieved during rehabilitation is especially low for individuals requiring more assistance from their therapist to stand and walk [15]. Electromechanical devices such as body weight-supported treadmills and treadmill-based robotic devices have been proposed to provide walking practice to non-ambulatory individuals during stroke rehabilitation [16, 17], though some research has not supported their use [18, 19]. A possible reason for the mixed findings is the suggestion that treadmill-based assisted gait training does not fully replicate the task-specificity of overground walking [20].

Powered robotic exoskeletons are a more recent technology developed to enable walking for anyone with lower extremity weakness, without the constraints of prior mechanical devices. These wearable robots strap around the torso and legs to control joint motion to automate overground walking and can be used independent of a treadmill or overhead harness system. Early research has demonstrated safe use of powered robotic exoskeletons for individuals with stroke, but few clinical trials have been conducted to determine the efficacy of using such devices; fewer still have compared exoskeletal gait training to standard physical therapy care during early stroke recovery and rehabilitation [21–23]. Additionally, no studies have yet explored the perception and experience of either individuals with stroke or physical therapists towards using powered robotic exoskeleton technology for rehabilitation, a necessary consideration when introducing technology into practice [24].

The present study is designed to examine the adoptability of a powered robotic exoskeleton for stroke rehabilitation by determining the efficacy and acceptability of exoskeleton-based gait retraining. More specifically, this mixed-methods trial aims to: 1) determine the efficacy of exoskeleton-based gait rehabilitation to improve walking ability, function, cognition, and quality of life; as well as 2) explore the experience and perception of using a powered robotic exoskeleton for rehabilitation from the perspective of patients with stroke and their physical therapists. It is hypothesized that exoskeleton-based gait rehabilitation will result in greater improvements in walking ability, function, cognition, and quality of life compared to usual physical therapy care.

## Methods

**Fig. 1:**
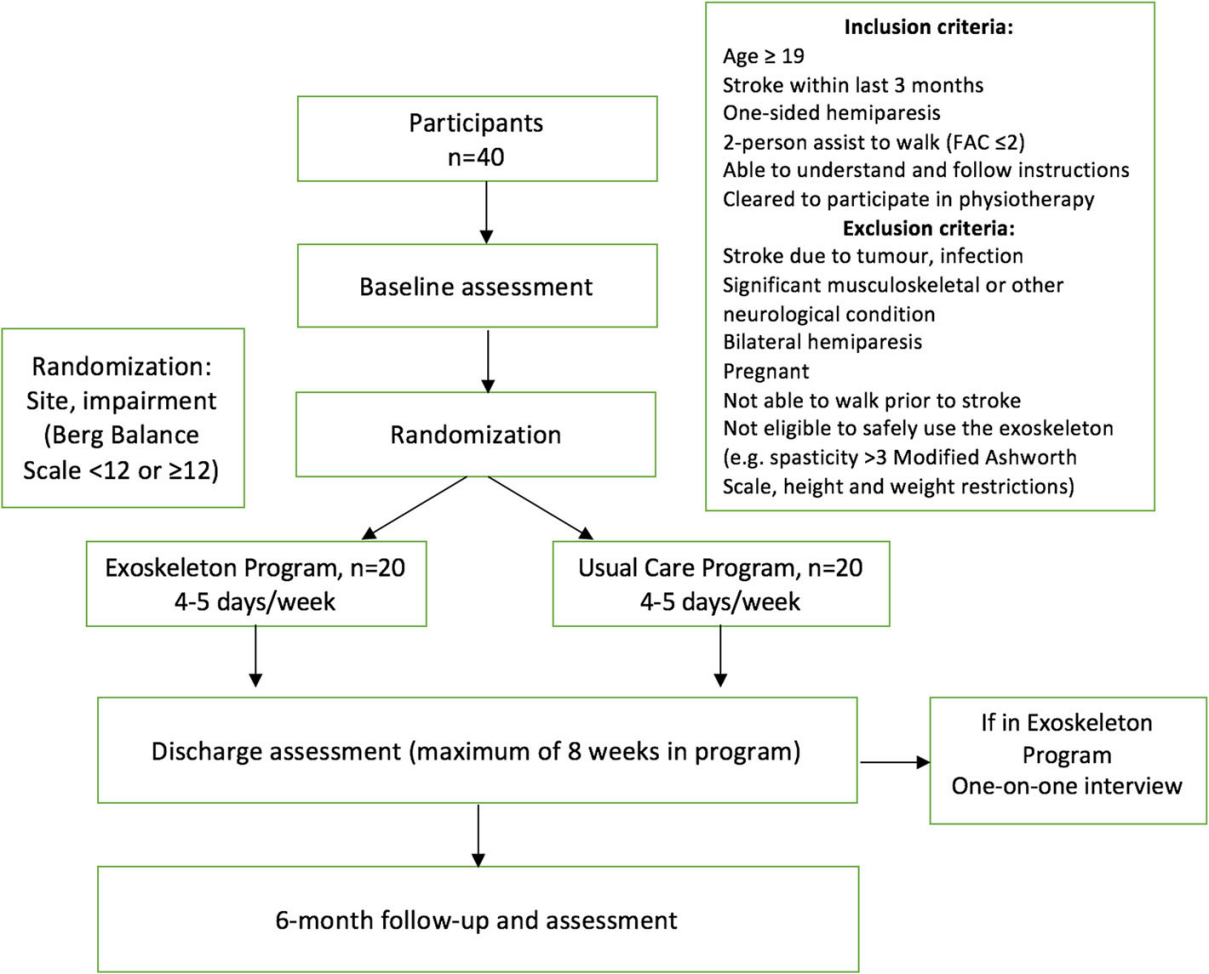
Flow diagram of mixed-methods study

This nested mixed-methods study will be comprised of a multi-center, parallel-group randomized, controlled trial (RCT) with an embedded qualitative study [25]. A flow diagram of the study procedures can be seen in Fig. 1. The methods for the quantitative and qualitative components are described here separately.

### Quantitative component: randomized, controlled trial

#### Setting

The RCT will be conducted at three rehabilitation hospitals, GF Strong Rehabilitation Centre (Vancouver, Canada), Glenrose Rehabilitation Hospital (Edmonton, Canada), and Parkwood Institute (London, Canada). Participants will be recruited from each respective inpatient stroke rehabilitation units over a period of up to three years.

#### Participants

Consecutive patients with subacute stroke admitted at each rehabilitation hospital will be identified by their treating physiatrists and therapists to be screened for eligibility by a member of the research team. Individuals will be included if they: 1) are within 3 months of stroke onset (ischemic infarct or intracerebral hemorrhage); 2) have one-sided hemiparesis; 3) are 19 years of age or older; 4) are able to understand and follow directions in English; 5) are able to communicate (verbal or physical yes/no indication); 6) are cleared to participate in physical therapy; and 7) require significant assistance (maximal assistance from one or two people) to walk. Individuals will be excluded from the study if they have: 1) a significant musculoskeletal or other neurological condition; 2) cardiovascular contraindications to exercise; 3) co-morbidities that would preclude activity; 4) or pain which is intolerably worsened with exercise. Individuals will also be excluded if they have any contraindications to using the robotic exoskeleton (pregnant, height/weight restrictions). The site coordinator at each hospital with obtain informed consent from potential trial participants.

#### Randomization

Participants will be randomized by the site coordinator after the baseline assessment at a one-to-one ratio to either the Exoskeleton group or Usual Care group using an online, third-party, permuted block randomization service (www.randomize.net, Interrand Inc., Ottawa, ON). As such, randomization will remain concealed until group allocation. Randomization will be stratified by site, to control for differences in standard of care (e.g., frequency and duration of physical therapy treatment, length of stay, rehabilitation and admission discharge timing, etc.). Participants will also be stratified by physical function, as baseline functioning is an independent predictor of outcomes such as community mobility and discharge destination [8, 26]. Specifically, participants will be stratified using their baseline Berg Balance Scale score at enrolment, as it has been shown to be correlated with improved walking ability after robot-assisted gait training [27]. A cut-off score of 12 will be used to stratify participants, based on a study [28] which identified a score of 12 at rehabilitation admission to be predictive of regaining unassisted walking after four weeks.

#### Exoskeleton device

The EksoGT powered robotic exoskeleton (Ekso Bionics, Richmond, California, USA) will be used to provide the experimental intervention for this study. This exoskeleton has bilateral motor-actuated joints at the hip and knee, as well as a spring-loaded articulation at the ankle to support toe-off and foot clearance during gait via a footplate. The EksoGT is able to power the user’s lower limbs in a walking pattern autonomously (without any active participation by the user), as well as with varied assistance to accommodate any force contribution by the user. The device software allows the therapist to control the degree of assistance, the parameters of gait (step height, step length, swing speed, etc.) and the automaticity of walking (how each step is triggered). These settings can be programmed to tailor the gait training to the individual to ensure active participation that is appropriately challenging. Guidelines for programming the device software to progress gait training with respect to robotic assistance are available in Additional file 1. The device does not provide balance support, and so the user is responsible for maintaining balance and shifting their weight appropriately.

#### Exoskeleton intervention (experimental)

Participants in the Exoskeleton group will have 75% of their standard physical therapy sessions replaced with exoskeleton-based gait rehabilitation. For example, 3 out of 4 weekly physical therapy sessions, or 45 min out of every 60-min session, will be dedicated to the exoskeleton intervention, reserving 25% of therapy time to be dedicated towards other goals. Participants in the Exoskeleton group will wear a powered robotic exoskeleton for their physical therapy sessions beginning after the baseline assessment to allow for repetitious stepping and walking practice from early in their rehabilitation stay. Training will be safely progressed, as tolerated, to reduce the amount of assistance provided by the exoskeleton and to increase the duration of continuous walking bouts. Guidelines for training progression can be seen in Table 1, and specific device programming is available in Additional file 1.

**Table 1:** Training progression for experimental group receiving exoskeleton intervention

Timing	Exoskeleton training guidelines
Week 1 (i.e. First 3–4 exoskeleton sessions)	- Require 30 min of upright time in exoskeleton, no set requirement for time in walking (expect approximately 10 min) - Aim for at least 250 steps per session - Familiarize with device, high assistance from therapist and robotics
Week 2 (i.e. 5th session and on)	- Require 15 min of walking time, of 30 min of upright time - Aim for 400 steps per session - Begin reducing assistance from therapist and robotics
Week 3 (i.e. 10th session and on)	- Require 20 min of walking time, of 30 min of upright time - Aim for 550 steps per session - Continue reducing assistance from therapist and robotics
Week 4 and beyond (i.e. beyond 15 sessions)	- Require 25 min of walking time, of 30 min of upright time - Aim for 700+ steps per session - Minimal assistance from therapist and robotics

**Fig. 2:**
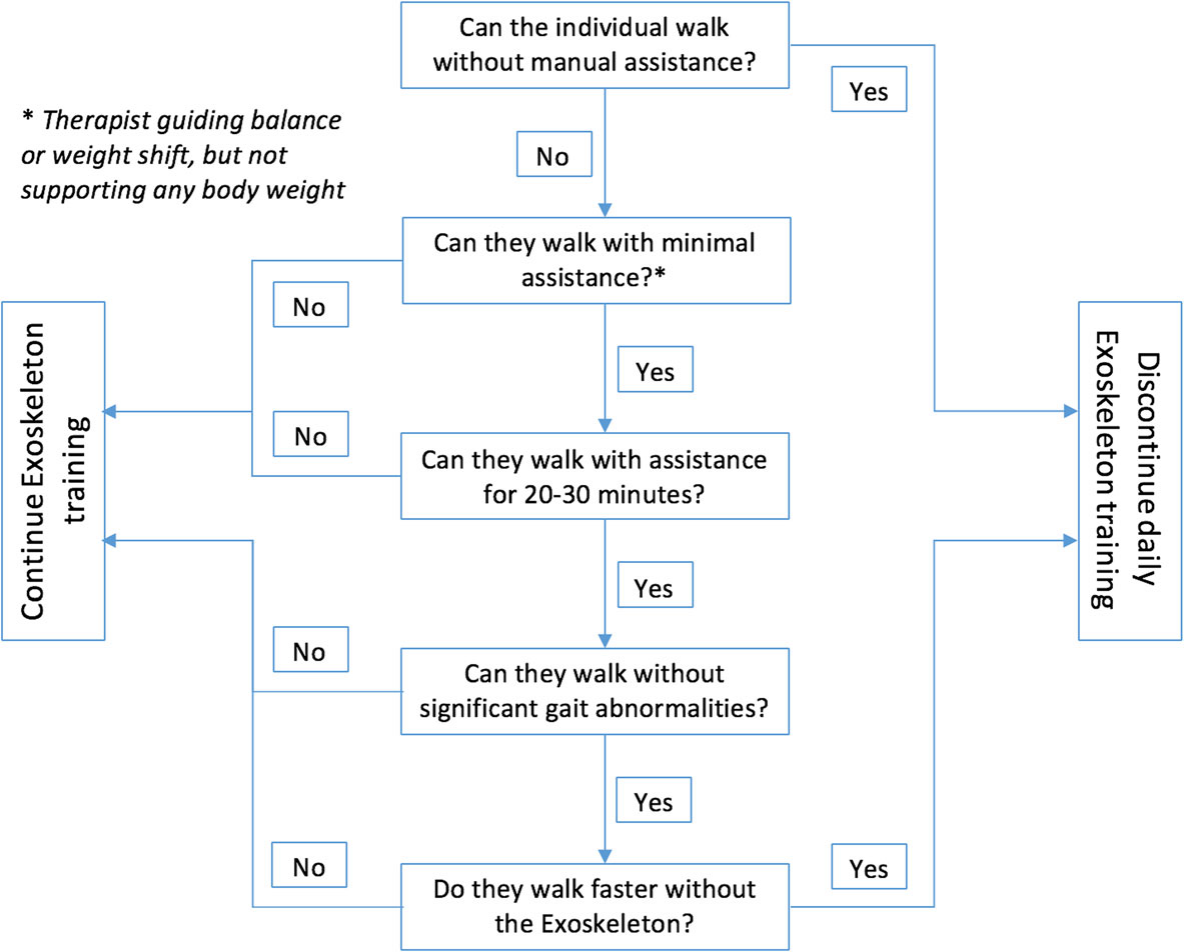
Algorithm to continue or discontinue daily exoskeleton training

An algorithm will guide clinicians in deciding when to discontinue daily exoskeleton training (Fig. 2), as it has been found that therapist-guided overground walking practice is equally or more effective than electromechanically-assisted gait for improving walking function once people with stroke are ambulatory [18, 19]. Once a participant reaches a functional threshold wherein they are able to walk for an extended period of time with only minimal assistance, therapists may begin to substitute daily exoskeleton training time to overground gait training. If a therapist chooses to fully discontinue use of the exoskeleton, they will still be required to focus on gait retraining for 75% of their weekly physical therapy time.

#### Usual care intervention (control)

Participants randomized to the Usual Care group will receive standard physical therapy care during their rehabilitation stay. Standard of care differs between sites, but typically involves 30–60 min physical therapy sessions, 4–5 days a week. No specific instructions will be given to therapists providing therapy in the Usual Care group, except that they cannot use the robotic exoskeleton. Generally, physical therapy during stroke rehabilitation is provided with patient-specific goals in mind, and typically places a large focus on mobility and gait training. Participants in both the Exoskeleton and Usual Care group will be monitored twice a week using an activity tracker (activPAL3 micro, PAL Technologies, Glasgow, UK) to observe the amount of upright standing and walking performed in the physical therapy sessions per group.

#### Evaluations

All participants will be assessed at recruitment (baseline), at discharge or after 8 weeks of the intervention, and at 6 months by an assessor who is blind to group allocation. The exoskeleton intervention will be discontinued after 8 weeks, and standard physical therapy will be provided to all participants beyond 8 weeks for whom it is deemed appropriate by their care team.

#### Primary outcome

The primary outcome will be walking ability, measured using the Functional Ambulation Category (FAC) [29]. This is a 6-item scale designed to classify the level of physical support required by subjects in order to walk safely over 10 ft, extending from 1 (unable to walk without the assistance of two people) to 6 (independent walking overground on uneven surfaces and on stairs). It has been shown to have good test-retest reliability and validity in the hemiparetic stroke population [30]. The FAC is also responsive to change within the first four weeks post-stroke and up to six months post-stroke [30]; unlike other walking measures of speed or distance, a value is assigned for the FAC even if the participant is not yet independent in walking.

#### Secondary outcomes

The secondary outcome measures will assess stroke impairment, walking performance (speed, endurance, daily step count), balance, cognition, and quality of life. The secondary outcome measures and schedule of data collection are listed in Table 2.

**Table 2:** Schedule of data collection

Study Procedures	Screening	Baseline evaluation	Post-intervention evaluation	Six-month evaluation
Timepoint	-T_1_	T_0_	T_1_	T_2_
Informed consent	+			
Inclusion/exclusion criteria	+			
Demographics		+		
Randomization		+		
Primary outcome measure				
Functional Ambulation Category [30]		+	+	+
Secondary outcome measures				
*Impairment*				
Fugl-Meyer Assessment (Lower extremity) [31]		+	+	+
*Functional*				
5-Meter Walk test [32]			(+)	(+)
6-Minute Walk test [33]			(+)	(+)
Berg Balance Scale [34]		+	+	+
activPAL mean step count (in PT)			+^a^	
Days to unassisted ambulation			+^a^	
activPAL daily step count over 4 days [35, 36]				+
*Cognitive*				
Montreal Cognitive Assessment [37]		+	+	+
*Quality of life*				
Patient Health Questionnaire [38]		+	+	+
36-Item Short Form Survey [39]		+	+	+
Adverse events screen			+^a^	+

PT: Physical therapy.

() Parentheses indicate that the outcome will be assessed if the participant is able to walk without physical assistance.

^a^indicates that the measure will be taken or monitored throughout the intervention period

#### Safety monitoring

All sites will report minor and serious adverse events that occur from baseline through to the 6-month follow-up. Two expert physiatrists will review reports outlining adverse events, if they arise, annually, to advise on trial continuation.

#### Sample size estimates

A total of 20 participants will be enrolled in each group (total same *n* = 40). This sample size was calculated using Stata Software (version 11, StataCorp, USA) and assumes a 2-point between-group difference in the Functional Ambulation Category at the end of the intervention [40], setting power at 80% and level of significance at 0.05 (2-sided). This calculation also assumes a standard deviation of 2.0 based on stroke inpatient FAC data from a study by Mehrholz et al. [30]. This between-group difference is realistic, given that participants are 2-person assist (score of 1 on the FAC) on enrolment, and it is expected that those in the Exoskeleton group will make greater improvements in walking ability (independent) compared to those receiving standard care (assistance or supervision required).

#### Statistical analyses

Descriptive statistics will be used to summarize data. An analysis of covariance (ANCOVA) will be performed to detect post-intervention differences between groups for the primary and secondary measures, using the respective baseline score as the covariate [41]. The significance level will be set at 0.05, and all statistical tests will be two-tailed. Participant data will be analyzed on an intention-to-treat basis, and any missing data will be assessed and analyzed as appropriate using multiple imputation [42]. For measures without a baseline score (i.e., 5-Metre Walk Test, 6-Minute Walk Test), an analysis of variance (ANOVA) will be employed. Confidence intervals (95% CIs) will be reported, where applicable.

### Qualitative component: interpretive description

This qualitative descriptive study will be conducted concurrently with the randomized controlled trial to determine the acceptability of the exoskeleton device for stroke rehabilitation from the perspective of patients with stroke and their physical therapists. The qualitative description methodology is useful when a straight description of phenomena is required, without the need for developing theory [43]. It is a rigorous methodology that provides a comprehensive summary of experiences and perceptions that is often used in health sciences research [43]. The qualitative design was informed by the COREQ (Consolidated criteria for reporting qualitative research) checklist [44], which will be used to report the qualitative findings.

#### Approach

This study will be based in a postpositivist paradigm [45], assuming that exoskeleton users will have individual yet relatively patterned experiences and perceptions of the device. According to this paradigm, conclusions that are drawn regarding the acceptability of using a powered robotic exoskeleton can be generalized to other stroke rehabilitation sites regardless of the social contexts of the researcher and participants of this study.

#### Participants

Participants randomized to the Exoskeleton group in the quantitative RCT will be recruited from all sites to participate in qualitative interviews. All participants who undergo at least five training sessions in the exoskeleton will be invited to participate, provided they are able to communicate fully (English proficiency, non-severe aphasia). It is expected that 10–15 participants with stroke will be thus be eligible and interviewed, during which time data sufficiency will be reached and identified themes would not need to be adjusted by further data collected [46, 47].

All physical therapists who have been fully trained to use the exoskeleton device and who provide the intervention for the RCT will be invited to participate in qualitative interviews. Five to 10 physical therapists are expected to be eligible and participate.

#### Procedures / data collection

Individual semi-structured interviews will be conducted in a private office with participants with stroke as well as with physical therapists from the RCT. Interviews will last approximately 30 min and will be conducted by the same researcher, whether in-person or by telephone, to maintain consistency. Interviews will be audio-recorded.

The semi-structured interview guides were developed by the lead author and reviewed by two physical therapists and two physiatrists (Additional file 2). Development of the interview guides was informed by the Unified Theory of Acceptance and Use of Technology (UTAUT) [48]. Questions explore user perspective towards the usage (fitting, duration, frequency, etc.) and utility (efficacy, perceived benefits, drawbacks, etc.) of the device.

#### Data processing and analysis

All interviews will be transcribed verbatim and analyzed using thematic analysis [49]. Transcripts will be read and re-read to develop ideas and interpretations about recurring, converging, and contradictory patterns. Once familiarized with the transcripts, raw data will be inductively coded by two investigators, then iteratively conceptualized into broad categories which will eventually be grouped into relevant themes to provide an understanding of how the exoskeleton is perceived by patients and therapists who use the device.

#### Trustworthiness

Drawing from the postpositivist criteria of credibility, transferability, dependability, and confirmability [50], various strategies will be employed to ensure the trustworthiness of this qualitative component of the study. Triangulation of multiple perspectives towards the exoskeleton by interviewing both individuals with stroke and physical therapists will promote the credibility of the qualitative findings. Furthermore, combining two research methods is another method of triangulation which will add depth and rigor to the study. The qualitative findings will provide context for the interpretation of the quantitative trial results.

Research reflexivity will support the transferability of the qualitative findings. By ensuring that the positioning of the authors and personal assumptions are accounted for in conducting the study and made known in reporting, readers will be able to determine the extent to which the findings can be generalized to their own context [50]. A reflexive journal will be kept in order to facilitate reflection on any assumptions, power differentials, and interpersonal dynamics that may arise during interviews that may influence data collection and analysis, which pertains to the dependability and confirmability of the qualitative methods [50].

Finally, negative case analysis and participant checking will enhance the credibility of the qualitative analysis. By exploring divergent perspectives during interviews and paying attention to opinions counter to the majority during analysis, we will develop a greater depth and understanding of the experience of using a powered exoskeleton for stroke rehabilitation. Bringing findings back to participants to ensure the analysis corresponds to their original account will help to ensure richness and accuracy of the findings. Synthesized analyzed data and resulting themes will be presented to participants in a document, written in non-scientific wording. Participants will be asked if the findings match their experience, and if they would like to change or add anything; any added data will be cross-referenced with existing codes and integrated into the analysis [51].

### Trial status

Participant recruitment began on 5 May 2017 for GF Strong Rehabilitation Centre, on 7 December 2017 for Glenrose Rehabilitation Hospital, and on 8 August 2018 for Parkwood Institute. Participant recruitment is ongoing and projected to be completed by December 31, 2020.

## Discussion

This mixed-methods study is the first to investigate the adoptability of an exoskeleton device in stroke rehabilitation by concurrently determining the efficacy and acceptability of an exoskeleton-based gait retraining program during early stroke recovery. At a time when powered robotic exoskeletons are continually being developed, refined, and manufactured, the findings of this study will provide guidance to clinicians as to whether such devices should be employed for early stroke rehabilitation, and for which outcomes.

Currently, there are only a handful of inpatient rehabilitation facilities across Canada that house a powered robotic exoskeleton for clinical or research purposes. We anticipate that recruitment may be affected by potential participants’ perception of the robotic device; some participants may decline participation because of the novel and intimidating nature of integrating robotics into treatment, while others excited for the device may be disappointed if they are randomized to the Usual Care group and may subsequently withdraw from the study. To account for these concerns, potential participants will be informed of the safety features and specific purpose of using the exoskeleton for the study, and those randomized to the Usual Care group will be offered an opportunity to trial the exoskeleton after their intervention period.

Compared to other studies of electromechanical devices and robot-assisted gait training in which the robotic intervention is rigorously performed several times a week for the entire duration of the intervention period, the current study presents a more realistic clinical intervention in which the exoskeleton use is integrated within standard physical therapy care and the frequency of exoskeleton use is reduced once a certain target in walking improvement is reached. Previous research showed that ambulatory individuals with stroke fare worse when confined to robotic or harness systems [17, 18], and thus our protocol will progress participants to activity without the robot once they are able. We anticipate that this method of exoskeleton-use will be more acceptable to therapists, as the algorithms presented for progressing the exoskeleton training or discontinuing use of the exoskeleton allows independence and clinical reasoning on the part of therapists. We also anticipate this will support a smooth translation of the research findings into clinical practice once findings are disseminated.

By also conducting qualitative interviews with participants and their therapists, a deeper understanding of the utility and potential limitations of powered robotic exoskeletons in today’s health services for stroke rehabilitation will be gained alongside the efficacy findings. Without the positive reception of the device, regardless of demonstrated effectiveness, new technology often goes unused [20, 52]. Furthermore, the rich data gained from exploration of participant and therapist experience of using an exoskeleton will be integrated with the quantitative findings to serve knowledge translation efforts at study completion, as the personal accounts will potentially elucidate how best to utilize the device in therapy with respect to timing, frequency, set-up, and duration.

This study has several limitations. Individuals with more severe stroke requiring greater assistance to walk often have other impairments or co-morbidities that may affect their prognosis, which may affect recruitment as well as the outcomes of this research study. Another limitation is the inability to blind the therapists or participants to the study intervention. Finally, there is a chance that the qualitative data may not reach saturation, given the number of eligible participants with this sample size.

## Supplementary information

**Additional file 1.** Guidelines for Progressing Participant in Exoskeleton Group.

**Additional file 2.** Qualitative Interview Guides.

## Data Availability

Not applicable.

## Abbreviations

ANCOVA: Analysis of covariance
ANOVA: Analysis of variance
CI: Confidence interval
COREQ: Consolidated criteria for reporting qualitative research
FAC: Functional Ambulation Category
PT: Physical therapy
RCT: Randomized, controlled trial
SD: Standard deviation
