# Supplementary material for: Exoskeleton for post-stroke recovery of ambulation (ExStRA): study protocol for a mixed-methods study investigating the efficacy and acceptance of an exoskeleton-based physical therapy program during stroke inpatient rehabilitation
Source: BMC Neurol. 2020 Jan 28;20:35. doi: 10.1186/s12883-020-1617-7 (PMC6988257; doi:10.1186/s12883-020-1617-7)

Appendix 1: Guidelines for Progressing Participant in Exoskeleton Group
To standardize the rehabilitation received by participants in the Exoskeleton Group to a degree, the following guidelines to progress a participant are provided:

Initial settings:

**Walk Mode**	First Step
**Step Length**	Individualized
**Step Height**	0.3-0.5”
**Lateral Target**	2
**Side Assisted**	Bilateral
**Level of Assistance**	Adapt

Ideal target settings for hemiparetic stroke:

**Walk Mode**	Pro Step+
**Step Length**	Individualized
**Step Height**	0.1”
**Lateral Target**	-1, -2
**Side Assisted**	Free leg on strong side
**Level of Assistance**	Fixed 0-50

There may be participants who improve rapidly during the course of the intervention (whether due to spontaneous recovery or from functional practice). If a participant is improving faster than expected, i.e. is able to perform longer bouts of walking with the exoskeleton, achieving higher steps, walking with minimal assistance with the device, it is permissible to advance the participant faster than the provided guidelines. Certain progressions can be made simultaneously (e.g. fixed assistance with longer duration of walking).

Definition of EksoGT settings:
Walk mode *(how each step is triggered)*
First Step: Each step manually triggered by therapist by pressing a button
Pro Step+: Steps are triggered by exoskeleton user (by weight shift onto stance side, hip flexion moment on swing side)
Step Length *(length of each assisted step)*
Step Height *(height of foot clearance during swing, in inches)*
Lateral Target (*amount of lateral weight shift, units set by manufacturer*)
Side Assisted (*side of assistance from robotics)*
Bilateral: Both legs receiving assistance from robotics
Free leg: Unilateral assistance from robotics
Level of Assistance *(amount of assistance received from robotics)*
Adapt: fluctuating assistance provided by robotics for each step as needed
Fixed (0-100): set maximum of assistance provided by robotics for any given step (%)

Algorithm for Progressing the Intervention:

<u>Progression of settings to create best quality, fully-assisted gait in exoskeleton</u>

This process may be completed as quickly as the first or second session with the participant in the device. Some of these target values may not need to be reached in order to create best quality gait, and will also be guided by the visual appearance of the gait cycle.

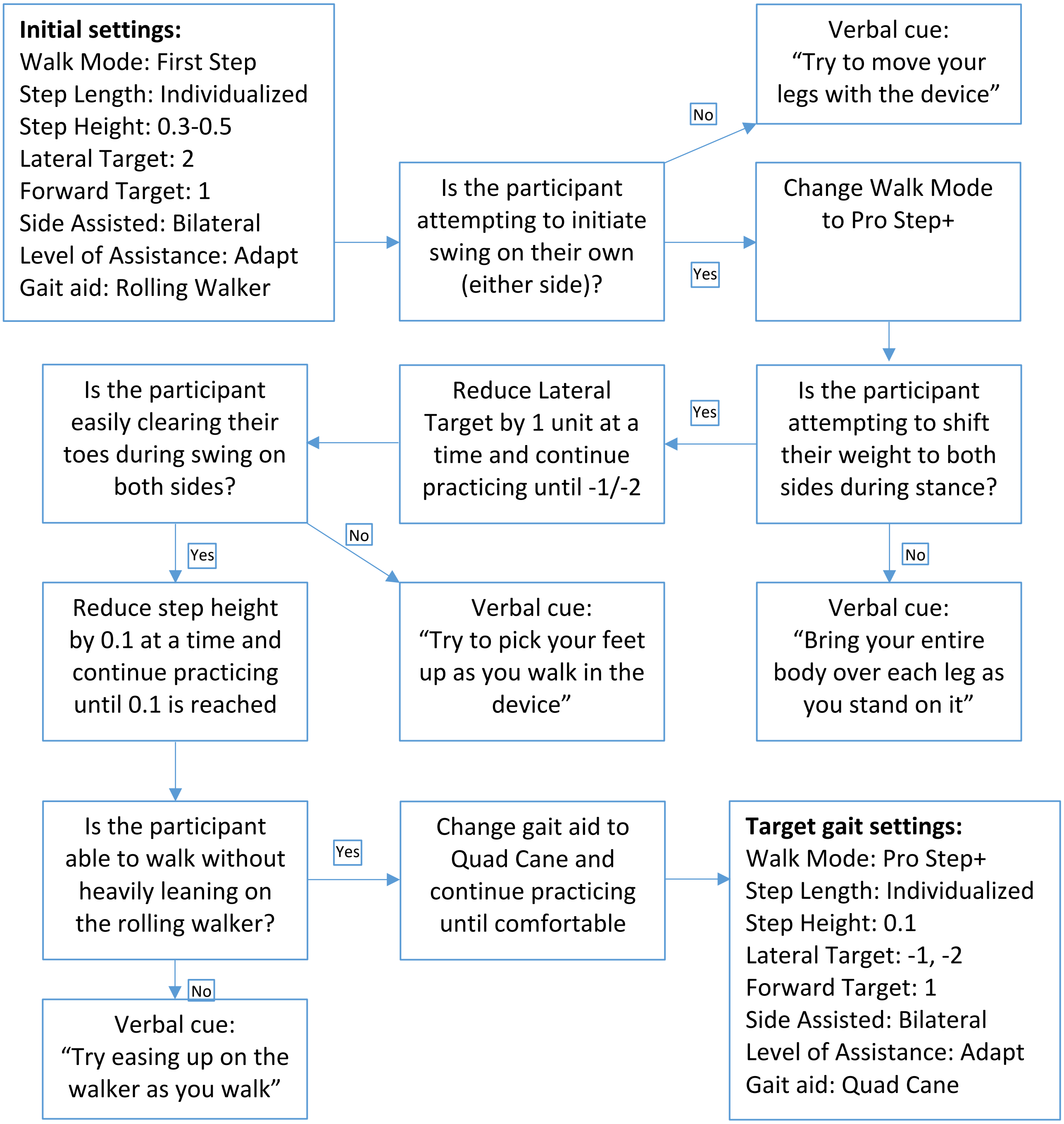

Algorithm to increase active participation and challenge while walking in exoskeleton

This flow chart should be used once the participant is comfortable walking in the device, requiring minimal verbal and physical guidance to maintain their balance in the device, to shift weight onto each stance limb, and to complete a swing. It may be very challenging for a participant to reach the final settings.

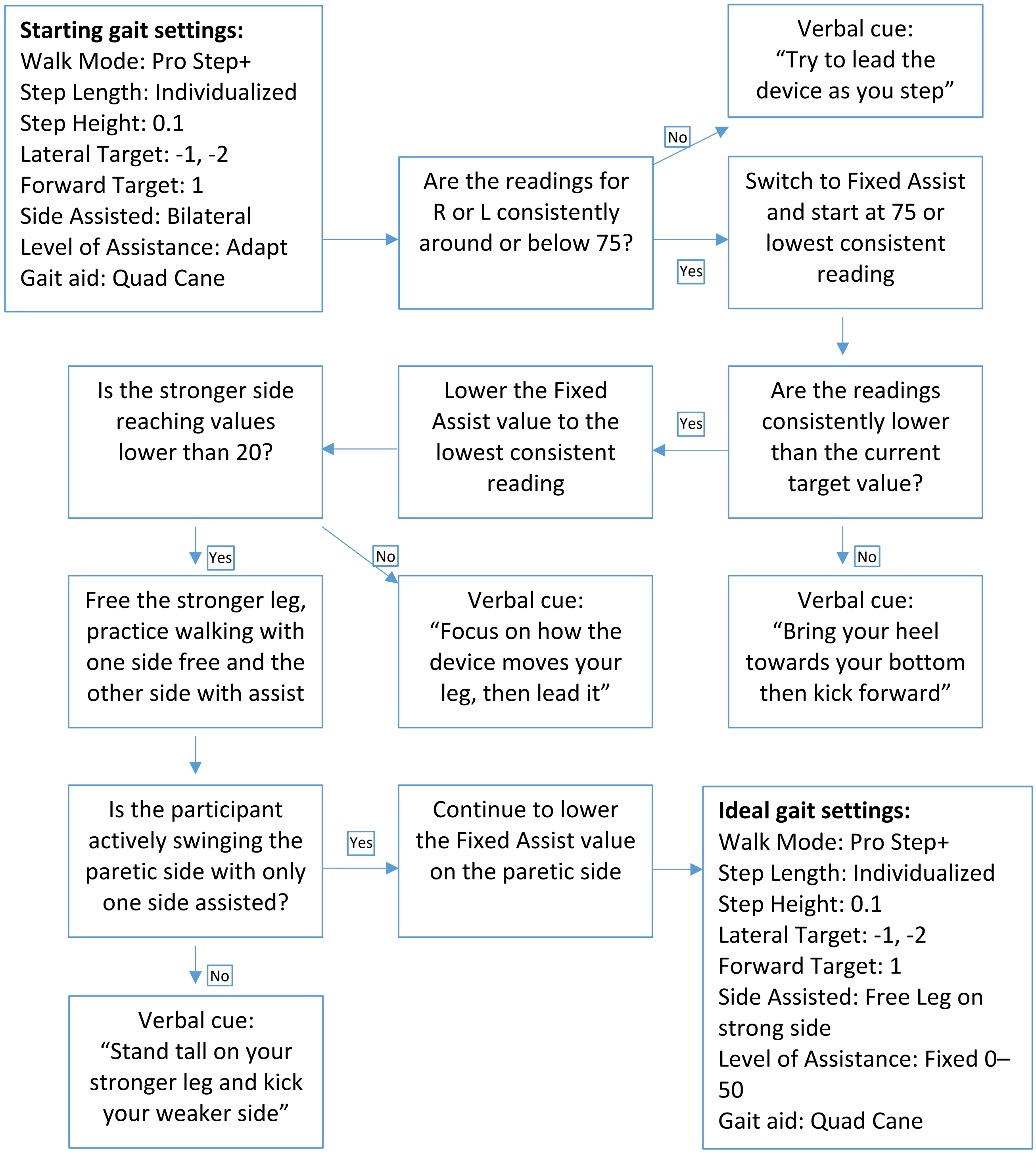

Supplement: Supplementary file 1 — Additional file 1. Guidelines for Progressing Participant in Exoskeleton Group. [file 12883_2020_1617_MOESM1_ESM.pdf]
