# Supplementary material for: Exoskeleton for post-stroke recovery of ambulation (ExStRA): study protocol for a mixed-methods study investigating the efficacy and acceptance of an exoskeleton-based physical therapy program during stroke inpatient rehabilitation
Source: BMC Neurol. 2020 Jan 28;20:35. doi: 10.1186/s12883-020-1617-7 (PMC6988257; doi:10.1186/s12883-020-1617-7)
Supplement: Supplementary file 2 — Additional file 2. Qualitative Interview Guides. [file 12883_2020_1617_MOESM2_ESM.pdf]

Appendix 2: Qualitative Interview Guides

Stroke Participant Interview Guide

[*ExStRA Study*]

How did you feel about your walking ability when you first came to GF Strong?

How do you feel about your walking ability now? (e.g., confidence, fear of falling, endurance)

What training did you receive on the Exoskeleton?

What did you like about using the exoskeleton for your stroke rehabilitation?

What didn't you like about using the exoskeleton for your stroke rehabilitation?

How were you progressed on the Exoskeleton?
Prompt: What did you like about this progression?
Prompt: How could it be improved?

What do you see as barriers to using the exoskeleton?
Prompt: with the technology (e.g., ease of use, noise, comfort)
Prompt: with the therapist (e.g., difficulties setting up, confidence in using device, attitude)
Prompt: with the current health care system (e.g., space, scheduling within the hour, access to equipment)
Prompt: yourself (client) (e.g., fatigue, pain, perceived assumptions and stereotypes, expectations)

What was easy about using the exoskeleton? What are the benefits to using the exoskeleton for stroke rehabilitation? (may query similar to 2)

What changes do you think should be made in the way the Exoskeleton was used in this study?

What are your thoughts on using the Exoskeleton beyond this trial, for other stroke patients?

Physiotherapist Interview Guide
[*ExStRA Study*]

How long have you been working in stroke rehabilitation?

What training have you received to use the Exoskeleton?

What did you think about the training you received?
Prompt: How could it be improved?
Prompt: What did you like about it?

Since you've been trained, what opportunities have you had to use the Exoskeleton with patients?
Prompt: What type of patients (diagnosis) did you use it with?

What has been your experience using the device in your regular practice?

What has been your experience using the device in the ExStRA study?

What are the barriers to using the exoskeleton for stroke rehabilitation?
Prompt: with the technology (e.g., ease of use, noise, quality of movement)
Prompt: with the patient (e.g., fatigue, pain, perceived assumptions and stereotypes)
Prompt: with the current health care system logistics (e.g., space, scheduling within the hour, access to equipment)
Prompt: yourself (therapist) (e.g. your training, your confidence in using the device, your own perceptions of the potential benefits)

What are the benefits to using the exoskeleton for stroke rehabilitation? (query similar to 4a)

How do you think using technology like the exoskeleton affects patient motivation, if at all?

How would you describe the ideal stroke patient for whom you think the Exoskeleton might be most suitable for? Why?
Prompt: What level of functioning would they be? (Consider cognitive and physical)
Prompt: At what point in their rehabilitation should they use it?

Describe how you have progressed the patient on the Exoskeleton?
Prompt: What did you like about it?
Prompt: How could it be improved?
Prompt: How many sessions does it take to see progression / improvement?

What changes do you think should be made in the way the Exoskeleton was used in this study?

How has exoskeleton-use affected how your work is organized?
Prompt: Is there sufficient support from your work setting to implementing exoskeleton training?

What are your thoughts on using the Exoskeleton beyond this trial, and in your everyday practice?
Prompt: Would you recommend exoskeleton device training to your clinical colleagues? All, or some?
Prompt: Would you recommend exoskeleton training to your patients?

Is there anything else you would like to add?
